# A Catecholaldehyde Metabolite of Norepinephrine Induces Myofibroblast Activation and Toxicity via the Receptor for Advanced Glycation Endproducts: Mitigating Role of l-Carnosine

**DOI:** 10.1021/acs.chemrestox.1c00262

**Published:** 2021-10-05

**Authors:** T. Blake Monroe, Ethan J. Anderson

**Affiliations:** † Department of Pharmaceutical Sciences and Experimental Therapeutics, College of Pharmacy, University of Iowa, Iowa City, Iowa 52242, United States; ‡ Fraternal Order of Eagles Diabetes Research Center, University of Iowa, Iowa City, Iowa 52242, United States

## Abstract

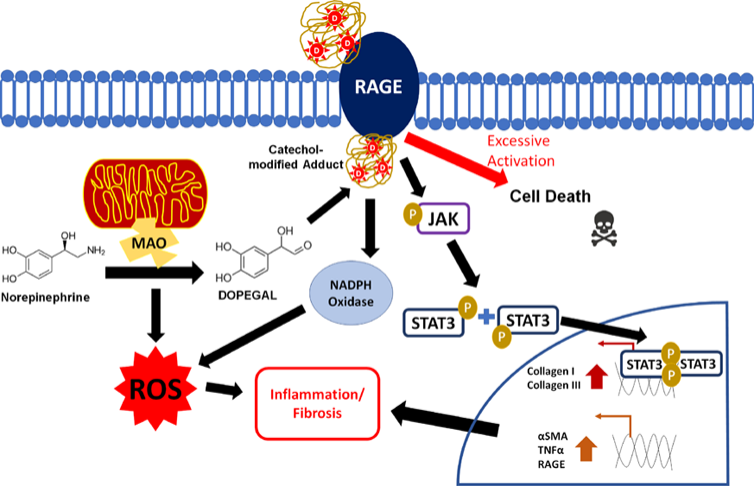

Monoamine oxidase (MAO) is rapidly gaining appreciation for its pathophysiologic role in cardiac injury and failure. Oxidative deamination of norepinephrine by MAO generates H_2_O_2_ and the catecholaldehyde 3,4-dihydroxyphenylglycolaldehyde (DOPEGAL), the latter of which is a highly potent and reactive electrophile that has been linked to cardiotoxicity. However, many questions remain as to whether catecholaldehydes regulate basic physiological processes in the myocardium and the pathways involved. Here, we examined the role of MAO-derived oxidative metabolites in mediating the activation of cardiac fibroblasts in response to norepinephrine. In neonatal murine cardiac fibroblasts, norepinephrine increased reactive oxygen species (ROS), accumulation of catechol-modified protein adducts, expression and secretion of collagens I/III, and other markers of profibrotic activation including STAT3 phosphorylation. These effects were attenuated with MAO inhibitors, the aldehyde-scavenging dipeptide l-carnosine, and FPS-ZM1, an antagonist for the receptor for advanced glycation endproducts (RAGE). Interestingly, treatment of cardiac fibroblasts with a low dose (1 μM) of DOPEGAL-modified albumin phenocopied many of the effects of norepinephrine and also induced an increase in RAGE expression. Higher doses (>10 μM) of DOPEGAL-modified albumin were determined to be toxic to cardiac fibroblasts in a RAGE-dependent manner, which was mitigated by l-carnosine. Collectively, these findings suggest that norepinephrine may influence extracellular matrix remodeling via an adrenergic-independent redox pathway in cardiac fibroblasts involving the MAO-mediated generation of ROS, catecholaldehydes, and RAGE. Furthermore, since elevations in the catecholaminergic tone and oxidative stress in heart disease are linked with cardiac fibrosis, this study illustrates novel drug targets that could potentially mitigate this serious disorder.

## Introduction

Myocardial fibrosis, the pathological accumulation of collagen in the myocardium, is the result of an excessive wound-healing response facilitated by fibroblasts becoming activated (i.e., myofibroblasts) in response to neurohumoral stimuli. In the myocardium, the transition of resident cardiac fibroblasts to a myofibroblast phenotype is marked by accelerated proliferation and increases in collagen deposition, expression of α smooth muscle actin (αSMA), and expression of proinflammatory chemo/cytokines.^1^ While it can be important in maintaining the integrity of an injured myocardium, extracellular matrix expansion also contributes to the pathogenesis of heart failure and arrhythmia by interfering with normal elasticity and electrical conductivity in myocardial tissue.^2^ Myocardial fibrosis is known to be associated with common metabolic conditions such as diabetes and obesity^3,4^ and is a prominent feature in the etiology of most cardiomyopathies.^1^ The molecular and cellular mechanisms underlying cardiac fibrosis are only partially understood, reflected in the current paucity of therapies specifically targeting this disorder.

While incomplete, the current understanding of cardiac fibrosis etiology involves a confluence of factors including oxidative stress and catecholaminergic and inflammatory signaling pathways.^5−8^ Monoamine oxidase (MAO) represents the major metabolic pathway for the breakdown of norepinephrine (NE) in the heart. MAO deaminates NE to produce ammonia, hydrogen peroxide, and the reactive aldehyde 3,4-dihydroxyphenylglycolaldehyde (DOPEGAL). This biogenic “catecholaldehyde” is substantially more cytotoxic and reactive than its parent catecholamines or any of its other known downstream metabolites.^9^ Although their role in neurotoxicity has been studied since the mid-1990s,^10^ catecholaldehydes have never been examined in the context of cardiovascular disease despite the rapidly emerging evidence supporting a pathogenic role for MAO in cardiomyopathy. For example, pathogenic roles for MAO in the adverse cardiac remodeling seen with diabetes^11^ and ischemia/reperfusion injury^12−14^ have recently been observed. Due to the reactivity of the electrophilic aldehyde and catechol moieties, DOPEGAL will readily react with nucleophilic species on biomolecules to form adducts.^15^ Adducts of biogenic aldehydes derived from oxidized sugars and lipids constitute advanced glycation products (AGEs) and advanced lipoxidation endproducts (ALEs), endogenous agonists of the receptor for advanced glycation endproducts (RAGE). RAGE has been shown to induce hallmarks of myofibroblast differentiation,^16^ including rapid fibroblast proliferation^17^ and collagen overproduction.^18^ Since RAGE is a pattern recognition receptor that binds a diverse array of ligands including glycated proteins^19,20^ and lipid-modified proteins,^21^ it seems plausible that RAGE is also activated by catechol-modified proteins.

While MAO activity in the myocardium has been examined as a source of oxidative stress in the myocardium, its capacity to promote phenotypic changes in resident cardiac fibroblasts has never been studied. Furthermore, most of MAO’s pro-oxidant activity is generally attributed to hydrogen peroxide generation and the pharmacological/toxicological effects of catecholaldehydes and their adducts are unknown. To address these knowledge gaps, we assessed MAO-mediated catecholaldehyde formation in cardiac fibroblasts and the potential for these reactive metabolites to mediate myofibroblast differentiation and collagen secretion. Our hypothesis was that MAO-generated oxidative metabolites stimulate fibroblast activation through a redox signaling pathway mediated by RAGE, representing a mechanistic link between increased sympathetic tone, oxidative stress, and the development of cardiac fibrosis in heart disease.

## Materials and Methods

### Primary Cardiac Fibroblast Preparation and Culture

Cardiac fibroblasts (CFs) were isolated from D1–D3 neonatal mouse hearts after digestion with enzymes from a commercially available kit (Pierce Primary Cardiomyocyte Isolation Kit) and differential plating technique. CFs were then cultured in Dulbecco’s DMEM/F12 media supplemented with 10% FBS and penicillin–streptomycin, and cultures were confirmed to be ≥99% CFs. All experiments were performed between passages 3–6. Since the FPS-ZM1 vehicle was DMSO and DOPEGAL adducts were synthesized with excess bovine serum albumin (BSA), the cells in all experimental groups received a vehicle treatment with equal amounts of BSA (3%) and DMSO (0.001%).

### Synthesis of DOPEGAL and DOPEGAL–BSA Adducts

Preparation of DOPEGAL was performed using a previously described method^22,23^ with some modifications. A 25 mM solution of NE (Sigma-Aldrich) in 10 mM potassium phosphate, sodium bisulfite buffer (pH 7.5) was combined with 100 μL (500 μg) of recombinant MAO (Corning Life Sciences, Tewksbury, MA) and incubated at 30 °C for 10 h with gentle shaking and exposure to oxygen. The reaction was terminated by ultracentrifugation at 100,000*g* for 30 min, and the supernatant was collected. Adducts of DOPEGAL–bovine serum albumin were synthesized by incubating aliquots of the supernatant in 3% BSA solution (Sigma-Aldrich) in 50 mM sodium pyrophosphate buffer (pH 8.8) overnight at 4 °C. Unreacted small molecules and salts were removed with a protein concentrator (Pierce, 88,513).

### Catechol-Modified Protein Extraction Using *m*-Aminophenylboronic Acid (*m*-APBA) Resin

**Figure 1:**
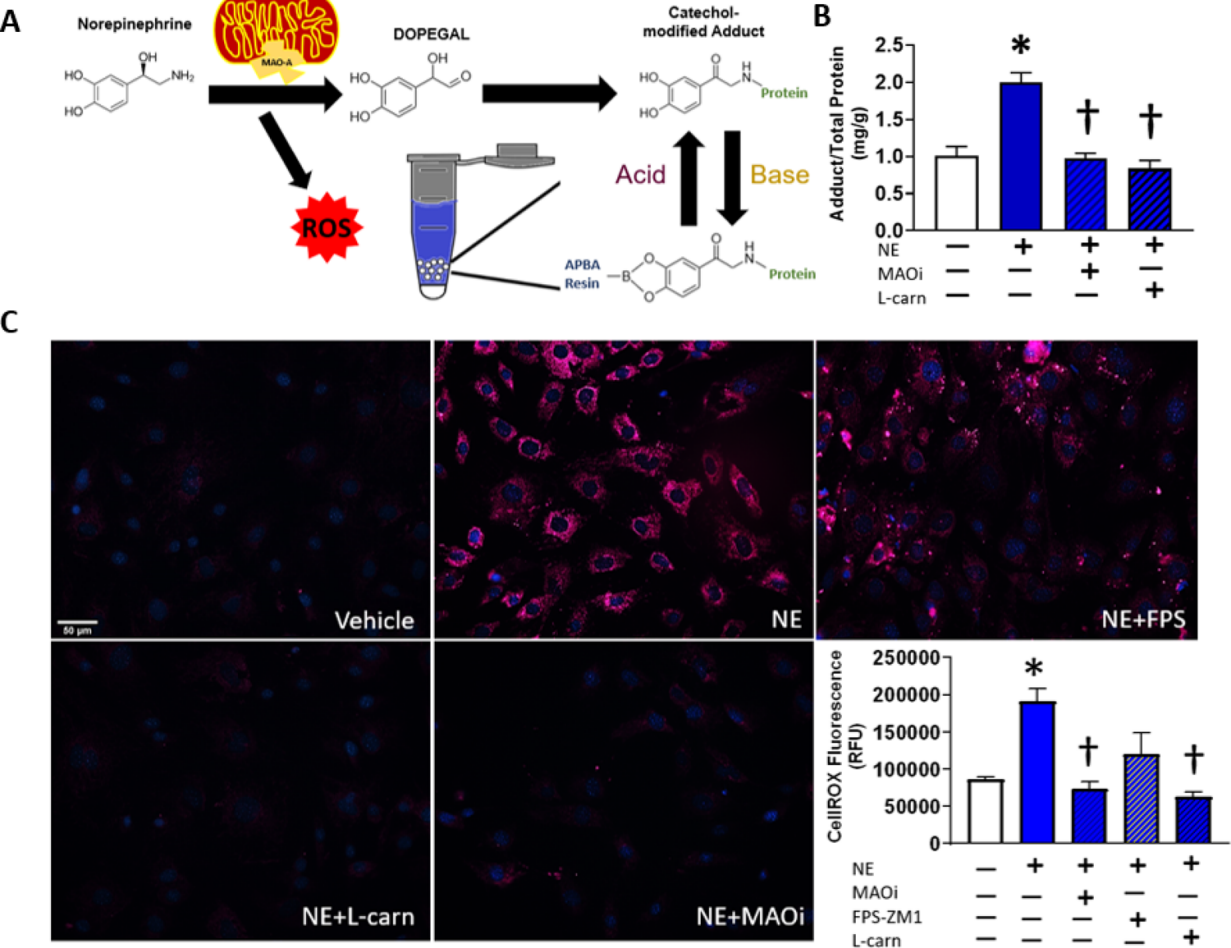
Catechol-modified adducts and oxidative stress in CFs following NE treatment. Reaction schematic showing norepinephrine (NE) is metabolized by MAO-A to produce DOPEGAL and H_2_O_2_, and then the catechol-modified protein adducts in cell lysates are captured using aminophenylboronic acid (APBA)-coated resin and quantified by a BCA assay (A). Cardiac fibroblasts (CFs) were treated for 96 h with NE (20 μM) alone or concurrently with MAOI (clorgiline + selegiline, 1 μM) or l-carnosine (10 μM), and the catechol-modified proteins from the lysate were isolated with APBA resin and quantified by a BCA assay (*n* = 3) (B). CFs were treated with NE (5 μM) for 48 h alone or concurrently with RAGE-antagonist FPS-ZM1 (223 nM), MAOIs, or l-carnosine as above, and the cytosolic ROS was visualized with a CellROX Deep Red reagent and then quantified using ImageJ and normalized to the nuclear Hoechst stain (*n* = 3) (C). *P < 0.05 versus vehicle control and †*P* < 0.05 versus the NE-treated group.

Catechol-modified protein adducts from the CF lysate were isolated using a modified version of a previously described method.^22,24,25^ As depicted in Figure 1A, the CF lysate (875 μg) was loaded onto *m*-APBA resin in 50 mM sodium pyrophosphate buffer (pH 8.8) and incubated overnight at 4 °C. The APBA agarose was then washed three times with a 1:1 ACN:50 mM sodium pyrophosphate buffer solution, once with a 5 mM sodium pyrophosphate buffer and once with ddH_2_O, centrifuging at 9000*g* and discarding the supernatant after each wash. Catechol-modified proteins were eluted from the resin by a 1:1 ACN:1% TFA solution for 1 h at room temperature. After the catechol-modified protein adducts from the lysate of the treated CFs were subjected to *m*-APBA pulldown, they were quantified using a BCA protein assay (Pierce).

### CF Proliferation

**Figure 2:**
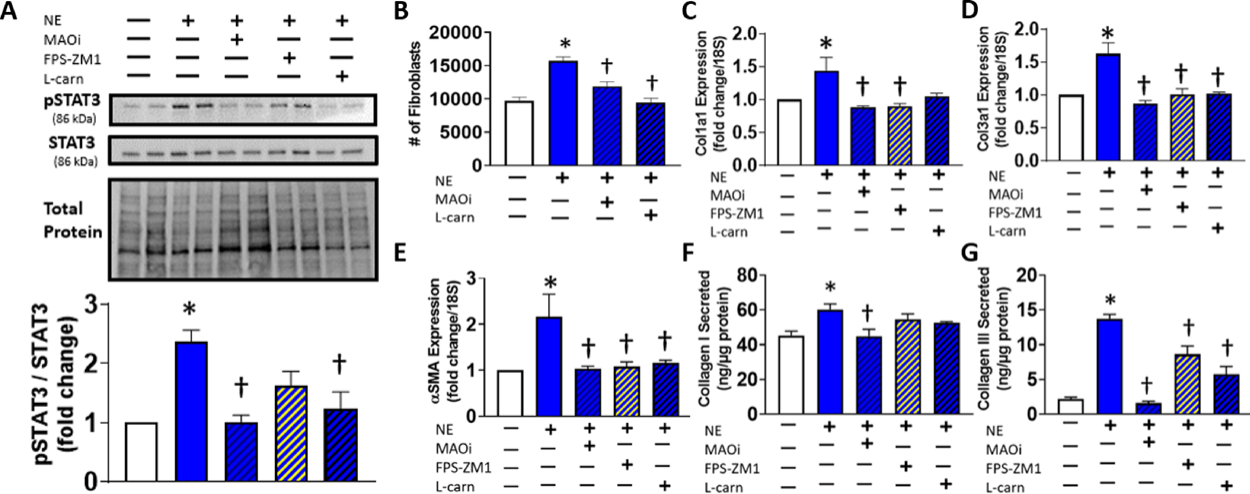
Redox-dependent effects of NE on CF activation and profibrotic phenotype. Representative immunoblot of STAT3 phosphorylation in the cell lysate from CFs treated for 6 h with NE (1 μM) alone or concurrently with MAOIs (clorgiline + selegiline, 1 μM), FPS-ZM1 (223 nM), or l-carnosine (10 μM) (A). CF proliferation after NE treatment for 48 h alone or concurrently with MAOIs or l-carnosine (B). Expression of Col1a1 (C), Cola1a3 (D), and α smooth muscle actin (E) in CFs treated for 6 h with NE alone or concurrently with MAOIs, FPS-ZM1, or l-carnosine. Collagen I (F) and III (G) secretion by CFs following treatment with NE alone for 96 h or concurrently with MAOIs, FPS-ZM1, or l-carnosine. *P < 0.05 versus vehicle control and ^†^*P* < 0.05 versus the NE alone group. Data are representative of *N* = 3–6 with a minimum of 2 replicates for each experiment.

Fibroblasts on a 96-well plate were treated as described (Figure 2). After treatment, the medium was removed and discarded, and the cells were assayed for proliferation using the CyQUANT Cell Proliferation Assay (Invitrogen). Per manufacturer’s instructions, following treatment, the cells were subjected to freeze/thaw and then assayed with a buffer containing a fluorogenic dye. The cells were lysed and incubated with a CyQUANT GR dye, and then the fluorescence was recorded at excitation/emission wavelengths of 485/528 nm using a plate reader (Synergy HTX, BioTek, Inc.). A standard curve was generated with serial dilutions of cell suspensions with known concentrations as determined by an automated cell counter (Countess II FL, Applied Biosystems). The total number of cells per well was determined by interpolating the unknown sample fluorescence values to the known values from the standard curve.

### ROS Visualization and Quantification in CFs

CFs cultured on glass-bottom, optically clear 24-well plates (CellVis) were preloaded with a 20 μM CellROX Deep Red reagent (Thermo Fisher) at 37 °C for 1 h. CFs were then washed with PBS and treated as described in the figure legends. After the treatment period, CFs were counterstained with a modified Hoechst stain (NucBlue Live ReadyProbes Reagent, Hoechst 33342). The treatment medium was replaced with a FluoroBrite DMEM medium (Gibco), and the cells were imaged with an EVOS Auto FL 2 Imaging System using light cubes with excitation/emission wavelengths of 357/447 nm and 628/685 nm for the modified Hoechst and CellROX dyes, respectively. The average intensity of CellROX fluorescence per cell was calculated using ImageJ (National Institutes of Health, v1.53) software.

### Immunoblot Analysis

Following treatments indicated in the figure legends, CF lysate (5 μg protein/lane) was loaded onto a 4–20% gradient acrylamide gel and subjected to SDS-PAGE. The proteins were then transferred to a PVDF membrane using a semi-dry transfer apparatus (BioRad). Total protein on the PVDF membrane was visualized using a No-Stain Protein Labeling Reagent (Thermo Fisher) and imaged on an iBright FL1000. The membrane was incubated with 5% BSA in TBS-Tween 20 to prevent nonspecific binding and then incubated with primary antibodies specific for RAGE (Santa Cruz, sc-365,154), phospho-STAT3 (Cell Signaling, 9145), and pan-STAT3 (Cell Signaling, 4904). Membranes were then washed and probed with appropriate secondary antibodies. The chemiluminescence signal was developed with a SuperSignal West Pico chemiluminescent substrate, western blots were imaged on the iBright CL1500 Imaging System (Invitrogen), and densitometric analysis was performed using their proprietary software (Invitrogen iBright Analysis Software). Following the immunoblot for phospho-STAT3, membranes were stripped using a Restore western blot stripping buffer, reblocked, and reprobed with the pan-STAT3 antibody.

### Quantitative Real-Time Polymerase Chain Reaction (qRT-PCR)

Treated CFs were lysed using a TRIzol reagent (Invitrogen), and mRNA was extracted following the manufacturer’s instructions. From each mRNA sample, cDNA was then synthesized by reverse transcription using SuperScript IV Reverse Transcriptase (Invitrogen). After addition of PowerTrack SYBR Green Master Mix and appropriate primers to targets of interest, cDNA was amplified on a QuantStudio3 Real-Time PCR system. Cycle threshold (*C*_t_) values were used to calculate gene expression relative to vehicle-treated controls using the 2^-ΔΔCt^ method and normalized to expression of 18*S*. The sequences for the forward and reverse primers used in the qRT-PCR experiments are listed in Table S1.

### Collagen Secretion

**Figure 3:**
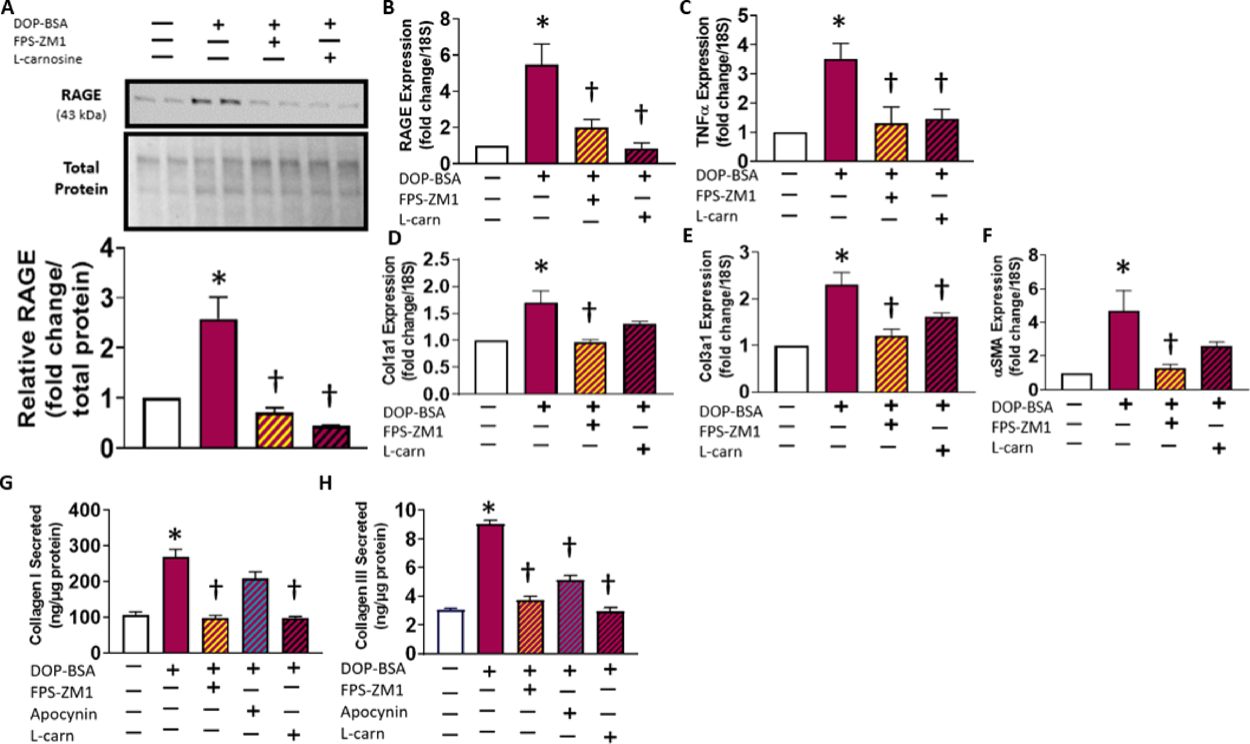
Proinflammatory, profibrotic effect of exogenous DOPEGAL–BSA adducts in CFs. The proinflammatory effect after treatment of CFs with DOPEGAL–BSA adducts (1 μM) alone or concurrently with FPS-ZM1 (223 nM) or l-carnosine (10 μM) on RAGE protein after 72 h (A) and mRNA (B) and TNFα expression after 48 h (C) are shown. Expression of Col1a1 (D), Cola1a3 (E), and αSMA (F) in CFs treated for 3 h with DOPEGAL–BSA alone or concurrently with FPS-ZM1 or l-carnosine. Collagen I (G) and III (H) secretion by CFs following treatment with DOPEGAL–BSA alone for 96 h or concurrently with FPS-ZM1, NOX inhibitor apocynin (2 μM), or l-carnosine. *P < 0.05 versus vehicle control and †*P* < 0.05 versus the DOPEGAL–BSA-treated group. Data are representative of *N* = 3–4 with a minimum of 2 replicates for each experiment.

After CFs were treated as described in the figure legends, the total protein in the media and CF lysate was collected and collagen was extracted from CFs using a modified form of a previously described method.^26^ This method employs an acidic pepsin solution consisting of gastric pepsin (1 mg/mL, Sigma-Aldrich) in 10 mM acetic acid to solubilize any potentially cross-linked collagen in the samples.^27^ The cells were washed with buffer, and collagen was extracted using the acidic pepsin solution for 2 h at 4 °C with rocking. The acidic solution was then collected, neutralized with 100 mM sodium hydroxide, and combined with the media of CFs treated as described in Figures 2 and 3. For the quantitative enzyme-linked immunosorbent assay (ELISA), serial dilutions of recombinant collagens I and III (Novus Biologicals) were prepared for standard curves and incubated, along with the CF samples, in a high-binding 96-well plate at 4 °C overnight. The plates were then washed and blocked with 5% BSA at 4 °C overnight. The samples were then incubated with primary antibodies for collagens I and III (Novus Biologicals) and then subsequently incubated with a horseradish peroxidase-conjugated secondary antibody. A solution of 10 μM Amplex Red (Thermo Fisher) was then added to generate the fluorophore resorufin that was measured at Ex/Em of 567/590 nm.

### Cytotoxicity Analysis

Living and dead CFs were quantified and visualized after treatments using the LIVE/DEAD Viability/Cytotoxicity Kit (Invitrogen). The LIVE/DEAD system relies on fluorophores generated from two reagents: calcein AM and ethidium homodimer-1 (EthD-1). Esterase activity in the cytosol of living cells produces a fluorophore from calcein AM in the green spectrum (Ex/Em = 494/517 nm). Generally unavailable to living cells with intact membranes, EthD-1 can bind to DNA in nonviable cells to produce a fluorophore in the red spectrum (Ex/Em = 528/617 nm). Briefly, treated CFs were loaded with both reagents and then imaged with an EVOS Auto FL 2 imaging system using FITC and TRITC light cubes to capture the excitation/emission wavelengths of 470/525 nm and 531/593 nm for calcein and EthD-1, respectively. The relative viability was also quantitatively assessed using LIVE/DEAD reagents. After loading, the fluorescence of the plated cells was measured using a SpectraMax M5 (Molecular Devices) plate reader at each of the fluorophore’s aforementioned Ex/Em maxima. The fluorescence values were then used to quantify cell death relative to groups of untreated cells, defined as 100% viable, and cells treated with 70% methanol for 45 min, defined as 100% dead.

### Statistical Analysis

Data were analyzed using a one-way ANOVA with multiple post hoc Tukey tests to compare differences between treatment groups. The analysis was performed using GraphPad Prism 9 (GraphPad Software, San Diego, CA). In the figures, data are presented as a mean ± standard error of the mean, with a *P* value less than 0.05 considered statistically significant.

## Results

### NE Catabolism by MAO Generates Catechol-Modified Adducts and Oxidative Stress in Primary Cardiac Fibroblasts

The aldehyde group on DOPEGAL has been shown to readily react with nucleophilic species on biomolecules, particularly amines, to form Schiff bases, which then undergo Amadori rearrangement to form catechol-modified adducts.^10^ In order to isolate catechol-modified adducts for quantification, we employed a method that we previously optimized and validated for extracting DOPEGAL-modified protein adducts from human myocardial tissues.^22^ This method uses an aminophenylboronic acid (APBA) resin to selectively bind catechol moieties under basic conditions. Captured adducts can then be released from the resin with acid and then quantified with a bicinchoninic acid (BCA) assay (Figure 1A). Exposure to NE substantially increased catechol adduct levels in primary murine cardiac fibroblasts (CFs), which were attenuated with monoamine oxidase inhibitors (MAOIs) clorgiline and selegiline (Figure 1B).

To probe the role of the aldehyde moiety in the chemistry of adduct formation as well as its relevance in promoting a profibrotic phenotype in CFs, we used l-carnosine in these experiments. l-Carnosine is an endogenous β-alanyl histidine dipeptide that is highly enriched to millimolar quantities in the heart, muscle, and brain^28−30^ and has a potent capacity to sequester and detoxify reactive carbonyl species.^31−34^l-Carnosine has been shown to form a stable product with the aldehyde metabolite of dopamine, DOPAL, and DOPEGAL.^35^ Concurrent treatment of NE with l-carnosine abrogated the formation of catechol-modified protein adducts (Figure 1B).

Given that the catecholaldehydes necessary to form protein adducts are generated alongside H_2_O_2_, we would expect an increase in cellular ROS to accompany protein adduct formation. Using the cytosolic ROS indicator CellROX,^36^ we found that NE increased cytosolic ROS in the CFs along the same time frame as catecholaldehyde formation (Figure 1C). This ROS is normalized through concurrent treatments of MAOIs and l-carnosine, but not RAGE antagonist FPS-ZM1.

### NE Induces a Profibrotic Phenotype in CFs via MAO and Downstream RAGE Signaling

NE is known to activate fibroblasts,^37−40^ but a role for MAO-mediated NE metabolites in this physiological process has never been explored. One pathway that putatively affects changes in collagen secretion involves phosphorylation of STAT3 by Janus kinase.^41,42^ When fibroblasts were stimulated with NE, there was a clear, corresponding phosphorylation of STAT3 with no accompanying change in the total STAT3 expression. Phosphorylation of STAT3 was eliminated with the inhibition of MAO activity and ROS formation (Figure 2A). NE stimulated an increase in CF proliferation that was also abrogated with concurrent MAOI or l-carnosine treatment (Figure 2B).

To assess the extent of profibrotic shift in CFs, we measured the expression of Col1a1 (Figure 2C), Col3a1 (Figure 2D), and α smooth muscle actin (αSMA, Figure 2E) genes in response to NE and again found that NE-induced up-regulation of profibrotic gene expression was blunted with concurrent treatment with MAOIs, l-carnosine, and FPS-ZM1. To further probe the functional consequences of the MAO-mediated effect of NE, we developed and validated a quantitative ELISA method based on a previously published method^26^ to determine the total amount of collagen I and III secretion by CFs, and these values were normalized to total cellular protein. Secretions of both collagens I (Figure 2F) and III (Figure 2G) were stimulated by NE treatment, and this secretion was abrogated by MAOIs, l-carnosine, and FPS-ZM1.

### Exogenous Catecholaldehyde Adducts Phenocopy MAO-Mediated NE Metabolites in CFs via RAGE Signaling

Chronic activation of RAGE has been shown to be involved in mediating the pathophysiological cardiac remodeling observed in a number of diseases, including heart failure, diabetes, and obesity.^3,43^ Putatively, RAGE signaling acts through phosphorylation of p38 MAPK and NF-κB activation,^44^ phosphorylation of SMAD2/3,^45^ and phosphorylation of STAT3^18^ to activate fibroblasts and increase the production of inflammatory factors. Given that the RAGE antagonist FPS-ZM1 attenuated the effect of NE on fibroblast activation, we next tested the hypothesis that CFs treated with exogenous (i.e., extracellular) catechol-modified protein adducts act as RAGE agonists, similar to glycated albumin. To investigate the pharmacological effects of catechol-modified adducts, we treated fibroblasts with exogenously synthesized DOPEGAL-modified bovine serum albumin. DOPEGAL–BSA-treated fibroblasts exhibited a nearly 3-fold increase in RAGE protein levels (Figure 3A), accompanied by significant up-regulations in RAGE (Figure 3B) and TNFα (Figure 3C) mRNA. Furthermore, DOPEGAL-adduct phenocopied NE treatment in CFs, resulting in increased expression of profibrotic markers Col1a1 (Figure 3D), Col3a1 (Figure 3E), and αSMA (Figure 3F) and secretion of collagen I (Figure 3G) and collagen III (Figure 3H). RAGE signaling putatively induces oxidative stress in cells through a variety of pathways including activation of NADPH oxidase (NOX), and this was observed in CFs stimulated with DOPEGAL–BSA adducts (Figure S1). Additionally, DOPEGAL adduct-induced increases in oxidative stress and profibrotic, proinflammatory signaling could be completely abrogated with FPS-ZM1 or l-carnosine and partially abrogated with the NOX inhibitor apocynin.

### Concentration-Dependent Cytotoxicity of DOPEGAL–BSA Adducts in CFs Is Mediated by RAGE Signaling

**Figure 4:**
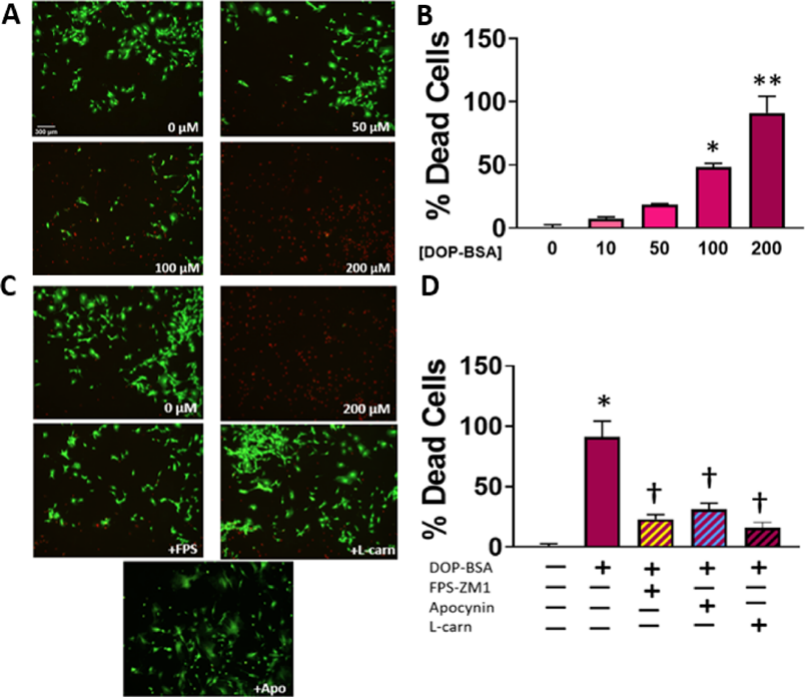
Toxicity of exogenous DOPEGAL–BSA adducts in CFs. Representative images (A) showing the dose-dependent toxicity of DOPEGAL–BSA in CFs using calcein AM (green) to stain living cells and ethidium homodimer-1 (red) to stain dead cells, and the overall ratio of live/dead cells was quantified at each DOPEGAL–BSA concentration (B). CFs were treated with a high dose of DOPEGAL–BSA adduct (200 μM) alone or concurrently with FPS-ZM1 (223 nM), apocynin (2 μM), or l-carnosine (100 μM), and the cells were imaged (C) and quantified (D) using the same technique. *P < 0.001 versus vehicle control, **P < 0.0001 versus vehicle control, and †*P* < 0.05 versus the DOPEGAL–BSA-treated group. Data are representative of *N* = 3–4 with a minimum of 2 replicates for each experiment.

RAGE activation is known to induce apoptosis in several cell types,^46−48^ including fibroblasts.^49^ Accumulation of ROS and activation of NF-κB, p38 MAPK, and caspase-3 are thought to play roles in RAGE-dependent apoptotic signaling.^46,50,51^ We next quantified and visualized the concentration-dependent effect of DOPEGAL–BSA on CFs and observed a dose-dependent cytotoxicity (Figure 4A,B), which was sensitive to RAGE signaling/oxidative stress inhibition (Figure 4C,D).

## Discussion

Catecholamine overload has long been known to be a major factor in the pathophysiology of cardiac fibrosis. Both chronic exposure to NE^52^ and pathologies associated with a hyperadrenergic state^53,54^ have been associated with severe fibrosis in the heart. Pathogenic mechanisms linking catecholamine excess to cardiac fibrosis are thought to be mediated largely via proapoptotic and/or profibrotic signaling driven by hyperstimulation of α-/β-adrenergic receptors in myocardial cells. However, a new paradigm of catecholamine pathogenicity in the heart has recently emerged, which is completely independent of canonical adrenergic signaling but rather dependent on the MAO-catalyzed oxidative metabolites formed from intracellular catecholamine metabolism. Indeed, MAO is now recognized as a nexus of oxidative stress that contributes to cardiac remodeling and dysfunction in diabetes,^11,55^ ischemia/reperfusion injury,^13,56^ and pressure overload.^57^ The present study provides evidence of a previously unappreciated redox signaling pathway that stems from the MAO-mediated metabolism of NE in primary CFs. Collectively, we have found that (1) NE stimulates ROS and catechol adducts in primary CFs in a MAO-dependent manner; (2) oxidative metabolites of NE stimulate proliferation, profibrotic gene expression, and collagen secretion via RAGE; (3) DOPEGAL–BSA adducts recapitulate the pharmacological effects of NE via RAGE at low concentrations; and (4) and DOPEGAL–BSA also causes cytotoxicity at high concentrations via RAGE. These findings have significant implications for extracellular matrix expansion under a broad range of cardiometabolic disease states.

MAO is tethered to the outer mitochondrial membrane and as such represents a substantial source of ROS in many cell types that have high mitochondrial content.^12^ In a recent study, we found that catecholaldehyde protein adducts were markedly increased in samples of atrial myocardium from diabetes patients as compared with age- and comorbidity-matched patients without diabetes.^22^ Furthermore, using APBA affinity chromatography, we found that mitochondria isolated from these atrial tissues contain >300 proteins modified by catechol adducts and that the total abundance of these modifications increase with NE exposure. We also observed that mitochondrial OXPHOS in the atrial myocardium is disrupted by NE in diabetes patients, where MAO activity is increased and levels of aldehyde dehydrogenase-2, a major aldehyde-detoxifying enzyme, are decreased. Thus, it is important to recognize that in cardiometabolic disease states (e.g., obesity/diabetes, heart failure, and atrial fibrillation) where oxidative stress is coming from multiple sources and is forcing a “bottleneck” in detoxification pathways, the findings of the present study are of particular importance. NE clearly stimulates catechol adduct formation and profibrotic phenotype in primary CFs, and this effect is mediated by MAO, as concurrent treatment with MAOIs and l-carnosine abrogates this effect (Figure 2).

Biogenic aldehydes, including DOPEGAL, react with nucleophilic species on proteins to create adducts including advanced glycation endproducts (AGEs) and advanced lipoxidation endproducts (ALEs).^58^ These adducts are now recognized to be very biologically active and mostly proinflammatory through their ability to activate RAGE. RAGE signaling has been implicated as a causal factor in many chronic diseases driven by oxidative stress^59−61^ including cardiac fibrosis.^43^ RAGE is not only activated by products derived from oxidative stress, RAGE signaling putatively induces oxidative stress via activation of NADPH oxidase (NOX), which leads to further AGE formation and thus amplifies RAGE activation in a “vicious cycle”.^62^ It is important to note that the formation of catechol-modified adducts coincided with an increase in overall cellular ROS (Figure 1). However, the fact that the increase in cellular ROS could be attenuated with MAOIs or l-carnosine, but not RAGE antagonist, suggests that the major route for NE-induced oxidative stress is through MAO activity and subsequent catecholaldehyde generation rather than NOX activation downstream of RAGE.

To our knowledge, the present study represents the first attempt to investigate the pharmacological activity of DOPEGAL-modified protein adducts (i.e., DOPEGAL–BSA), and our findings indicate that they occur through activation of RAGE. Inhibition of RAGE significantly blunted both NE- (Figure 2) and DOPEGAL–BSA-induced (Figure 3) expression of profibrotic factors and secretion of collagen in primary CFs. Taken with observed NE-induced phosphorylation of STAT3, a known downstream target of oxidative stress and RAGE signaling, the results of these experiments indicate a potential role for DOPEGAL and DOPEGAL-modified protein adducts in fibroblast activation through RAGE signaling. Although more work is clearly needed to better characterize the identity and reactivity of catechol-modified adducts, a recent structural study may help explain how DOPEGAL modification could make albumin a RAGE ligand.^21^ Modification of basic amino acids, namely, lysine, with a neutral catechol group, would make nearby negatively charged side chains available to bind to positive species in the V domain of RAGE. As discussed above, our group has identified potential targets for catechol adduct modification in the human heart mitochondria,^22^ and proteomics analysis is currently ongoing to identify and characterize catecholaldehyde–protein adducts in multiple other tissue/cell types, including primary CFs.

To conclude, our study provides evidence that NE is capable of activating and inducing a profibrotic phenotype in CFs via a redox signaling pathway that is completely independent of canonical α- or β-adrenergic signaling but is dependent on MAO and RAGE. Given that MAO has been found to be up-regulated in the heart with numerous cardiometabolic disorders, these findings indicate possible contributing roles for oxidative metabolites formed from MAO-mediated NE metabolism in extracellular matrix expansion and the pathogenesis of cardiac fibrosis. Ultimately, a more detailed understanding of how MAO affects adverse cardiac remodeling in vivo could potentially inform therapeutic strategies by identifying druggable targets (i.e., detoxifying biogenic aldehydes, inhibiting MAO, and antagonizing RAGE).

## Supporting Information Available

Table of primers used in RT-PCR, images of oxidative stress in DOPEGAL–BSA stimulated CFs (Figure S1), and control experiments for gene expression effects in CFs treated with inhibitors/antagonists and l-carnosine (Figure S2) (PDF)
