# Supplementary material for: A Catecholaldehyde Metabolite of Norepinephrine Induces Myofibroblast Activation and Toxicity via the Receptor for Advanced Glycation Endproducts: Mitigating Role of l-Carnosine
Source: Chem Res Toxicol. 2021 Oct 5;34(10):2194–201. doi: 10.1021/acs.chemrestox.1c00262 (PMC8527521; doi:10.1021/acs.chemrestox.1c00262)
Supplement: Supplementary file 1 — tx1c00262_si_001.pdf [file tx1c00262_si_001.pdf]

Supplemental Information for tx-2021-001364

A catecholaldehyde metabolite of norepinephrine induces myofibroblast activation and toxicity via RAGE: Mitigating role of L-carnosine

T. Blake Monroe[1] and Ethan J. Anderson[1, 2]

[1]Department of Pharmaceutical Sciences and Experimental Therapeutics, College of Pharmacy, [2]Fraternal Order of Eagles Diabetes Research Center, University of Iowa, Iowa City, IA

<u>TABLE OF CONTENTS</u>

p. S2- Supplemental Table 1: Forward and reverse primers

p. S3- Supplemental Figure 1: Oxidative stress in fibroblasts following treatment with DOPEGAL-BSA

p. S4- Supplemental Figure 2: Effect of MAOi's, RAGE antagonist and L-carnosine on inflammatory and pro-fibrotic gene expression in fibroblasts

Supplemental Table 1. Forward and Reverse Primer Sequences

Target		Forward Primer	Reverse Primer
Fibrotic	Col1a1	GGTCCTCGTGGTGCTGCT	ACCTTTGCCCCTTCTTTG
	Col3a1	AGGCTGAAGGAAACAGCAAA	TAGTCTCATTGCCTTGCGTG
	αSMA	GTGACATCGACATCAGGAAAGA	GATCCACATCTGCTGGAAGG
Inflammatory	TNFα	GGTGCCTATGTCTCAGCCTCTT	GCCATAGAACTGATGAGAGGGAG
	RAGE	GCATCAGGGTCACAGAAACC	ATTGGGATGGAATGTGGGGG
Housekeeping	18S	AGAGCGGGTAAGAGAGGTGT	GTCGGGGTCCGACAAAACC

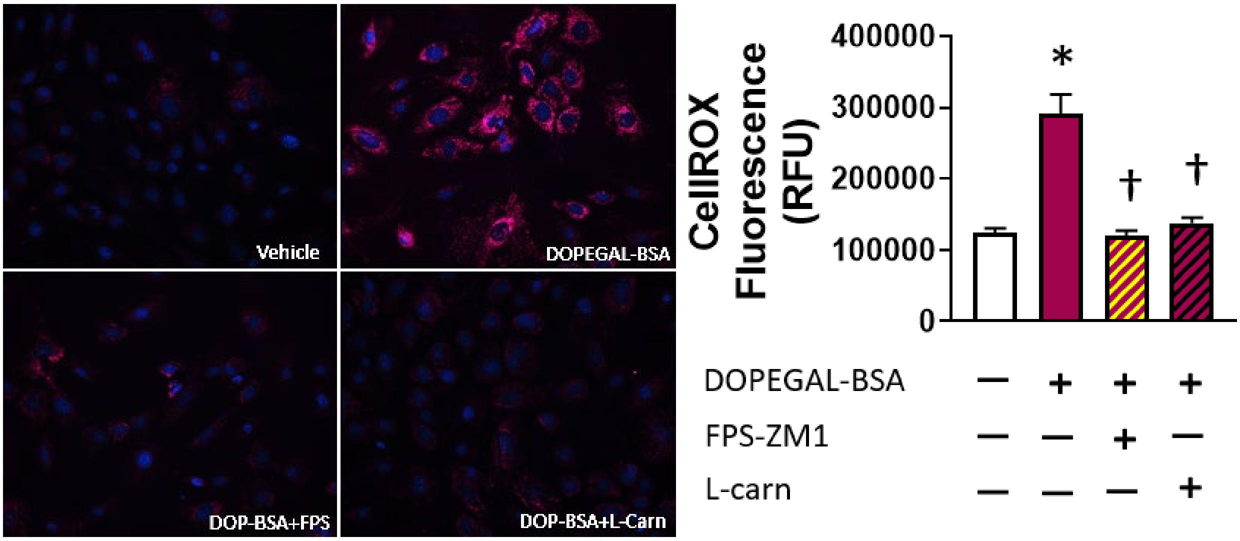

Supplemental Figure 1. Oxidative stress in fibroblasts following treatment with DOPEGAL-BSA.

CFs were treated with DOPEGAL-BSA adducts (10 μM) for 48 hours alone or concurrently with RAGE-antagonist FPS-ZM1 (223 nM) or L-carnosine (10 μM) and cytosolic ROS was visualized with CellROX Deep Red Reagent and then quantified using ImageJ and normalized to nuclear Hoechst stain (n=3)

* $P < 0.05$ versus vehicle control, † $P < 0.05$ versus DOPEGAL-BSA treated group.

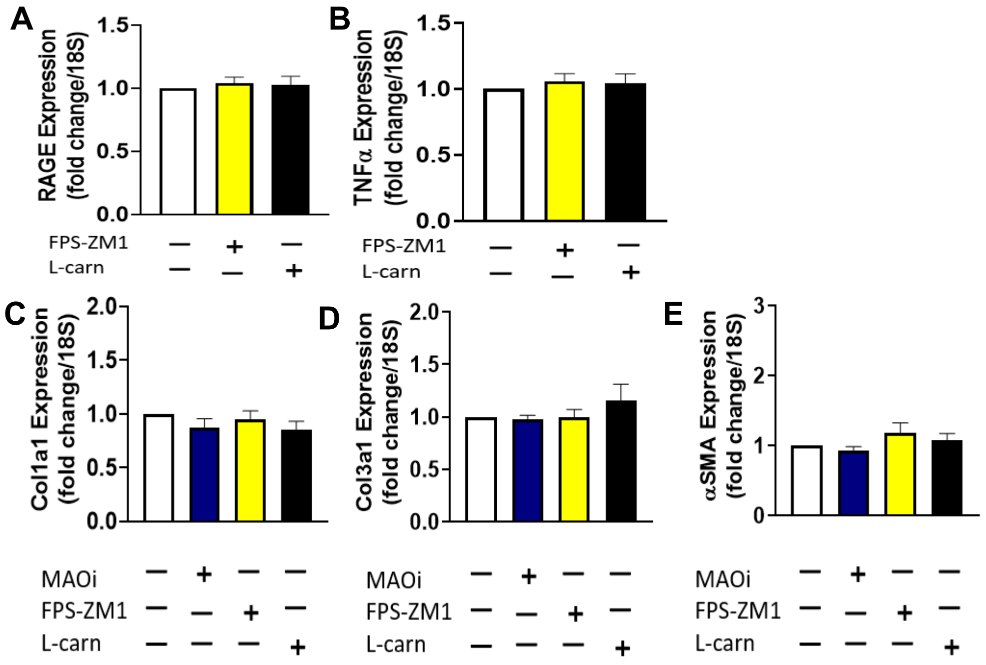

Supplemental Figure 2. Effect of MAOi's, RAGE antagonist and L-carnosine on inflammatory and pro-fibrotic gene expression in fibroblasts.
Gene expression of RAGE (**A**) and TNFα (**B**) in CF's treated for 48 hours with FPS-ZM1 or L-carnosine. Expression of Col1a1 (**C**), Cola1a3 (**D**), and α smooth muscle actin (**E**) in CF's treated for 6 hours with MAOi's, FPS-ZM1 or L-carnosine. Data are run in triplicate and representative of at least 3 independent experiments. $P > 0.05$ versus vehicle control for all groups.
